# Angiogenesis in adenosquamous cancer of pancreas

**DOI:** 10.18632/oncotarget.21319

**Published:** 2017-09-27

**Authors:** Nicola Silvestris, Katia Danza, Vito Longo, Oronzo Brunetti, Livia Fucci, Antonella Argentiero, Angela Calabrese, Ivana Cataldo, Roberto Tamma, Domenico Ribatti, Stefania Tommasi

**Affiliations:** ^1^ Medical Oncology Unit and Scientific Directorate, IRCCS Istituto Tumori “Giovanni Paolo II”, 70124, Bari, Italy; ^2^ Molecular Genetics Laboratory, IRCCS Istituto Tumori “Giovanni Paolo II”, 70124, Bari, Italy; ^3^ Medical Oncology Unit, Hospital “S. G. Moscati” of Taranto, 74010, Taranto, Italy; ^4^ Medical Oncology Unit, IRCCS Istituto Tumori “Giovanni Paolo II”, 70124, Bari, Italy; ^5^ Histopatology Unit, IRCCS Istituto Tumori “Giovanni Paolo II”, 70124, Bari, Italy; ^6^ Radiology Unit, IRCCS Istituto Tumori “Giovanni Paolo II”, 70124, Bari, Italy; ^7^ ARC-Net Research Centre, University and Hospital Trust of Verona, 37134, Verona, Italy; ^8^ Department of Basic Medical Sciences, Neurosciences and Sensory Organs, University of Bari Medical School, 70124, Bari, Italy; ^9^ IRCCS Istituto Tumori “Giovanni Paolo II”, 70124, Bari, Italy

**Keywords:** angiogenesis, adenosquamous cancer of pancreas, pancreatic ductal adenocarcinoma, miRNA, micro vascular density

## Abstract

Adenosquamous carcinoma of the pancreas (ASCP) is an uncommon variant of exocrine pancreatic malignancies, characterized by a histological admixture of adenomatous and squamous cell elements. This cancer is characterized by a poorly differentiated histology and a poorer clinical outcome compared to pancreatic ductal adenocarcinoma (PDAC). Unlike PDAC, that is characterized by a low microvascular density (MVD) and collapsed vasculature, no data are available about angiogenesis in ASPC. Immunohistochemical evaluation of MVD and trypatse-positive mast cells (MCs) were performed on a single case of ASCP compared to PDAC. Moreover, the levels of angiopoietin-1 and -2 (Ang-1, Ang-2), receptor tyrosine kinase with immunoglobulin and epidermal growth factor homology domain-2 (Tie-2), vascular endothelial growth factor A (VEGFA), hypoxia-inducible factor 1 alpha (HIF1A), miR-21-5p, miR-181a-5p, miR-122-5p, and miR-27a-3p were evaluated by real-time PCR. Higher number of tryptase-positive MCs and MVD are observed in the ASCP case compared to PDAC one. Lower levels of miR-122-5p and higher expression of VEGFA, HIF1A and Ang-2 genes were observed in ASCP. Furthermore, lower Ang-1 and Tie-2 transcript levels and higher increases of miR-21-5p, miR27a-3p and miR-181a-5p levels were found in the rarest form of pancreatic carcinoma. Our data demonstrate an important angiogenic activity in ASCP with a putative role of miR-21-5p, miR-181a-5p, miR-122-5p and miR-27a-3p in the regulation of this process.

## INTRODUCTION

Angiogenesis is a necessary condition for tumor growth and progression. In the absence of a blood supply which ensures the delivery of nutrients, tumors will not grow beyond 100-200 μm in diameter [1]. Among angiogenic factors, the vascular endothelial growth factor A (VEGFA), angiopoietin-1 (Ang-1) and angiopoietin-2 (Ang-2) emerged as the main regulators of this process with target specificity for endothelial cells (ECs). Binding to their endothelial receptors, these factors are responsible for proliferation, migration and survival of ECs but also for the integrity and maintenance of the vascular network [2, 3]. The involvement of epigenetic mechanisms in the regulation of angiogenesis has been previously described [4]. MicroRNAs (miRNAs) are small, non-coding RNAs that control several molecular pathways through the regulation of gene expression at the post-transcriptional level [5]. Due to their oncogenic and oncosuppressor properties, miRNAs can act as pro-angiogenic or anti-angiogenic elements during the formation of a novel blood vasculature [4]. A pivotal role in pancreatic ductal adenocarcinoma (PDAC) angiogenesis has been described for miRNAs [6]. PDAC is one of the first leading cause of cancer-related death worldwide that account for 85%–90% of all pancreatic tumors [7]. It is characterized by a fibro-inflammatory response that is responsible of high intratumoral pressure and solid stress causing vascular strickness and hypoxia. Hypoxic environment induces VEGFA, Ang-2 and other cytokines to attract macrophages, resulting in foci of angiogenesis in the peripheral areas of the tumor. However, the low microvascular density (MVD) and the collapsed vasculature makes ineffective the anti-angiogenic treatments in several clinical trials [8].

Unlike PDAC, it is not well known when the focus is restricted to the adenosquamous carcinoma of the pancreas (ASCP) that represents one of the less frequent histology of pancreatic cancer with an incidence of 3.06% [9]. So far, limited data on ASCP are available only from small, single-institution or retrospective studies. Given the pivotal role described for miRNAs in angiogenic cascades of several tumors [10] including PDAC [6], our aim was to explore the angiogenic pattern and its epigenetic regulation in ASCP. In this rare malignancy with few chemotherapeutic approaches, the characterization of the angiogenesis cascade may offer insight into potential biological therapeutic and diagnostic targets. To our knowledge, no data are available about the angiogenesis in ASCP. For this reason, we explore the transcript levels of Ang-1, Ang-2 and their receptor tyrosine kinase with immunoglobulin and epidermal growth factor homology domain-2 (Tie-2), VEGFA and hypoxia-inducible factor 1 alpha (HIF1A) in ASCP and comparing the expression levels of these genes with those observed in PDAC. Furthermore, to address our hypothesis of miRNAs involvement in ASCP angiogenesis, we also analyzed the levels of miR-21-5p, miR-181a-5p, miR-122-5p and miR-27a-3p. To support our data, we compared the microvascular characteristics of ASCP and the composition of inflammatory infiltrate in terms of tryptase-positive mast cells (MCs) with those observed in a PDAC case.

## RESULTS

TarBase v.6 [11] counseling emphasized Tie-2 gene as validated target of miR-21-5p. Furthermore, miRWalk v.2 database [12] highlighted Ang-1 as a predictive target of miR27a-3p and miR-181a-5p, whereas VEGFA, Ang-2 and HIF1A genes as predictive targets of miR-122-5p. The expression of miRNAs and VEGFA, HIF1A, Ang-1, Ang-2 and Tie-2 genes were performed by real-time PCR (M&M).

**Figure 1:**
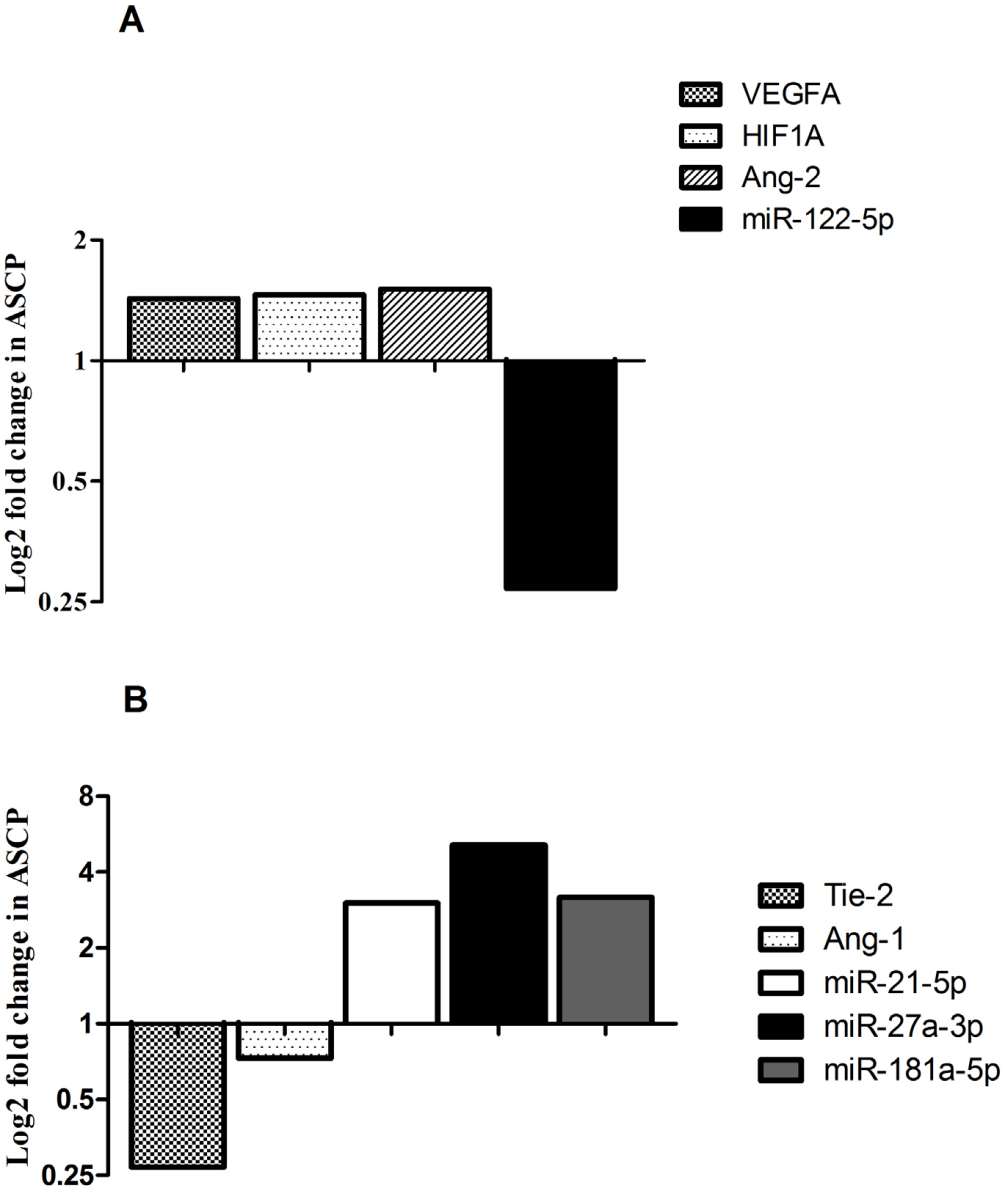
Bar graphs show log2 fold changes of differentially expressed miRNAs and genes between ASCP and PDAC cases Higher VEGFA, HIF1A, Ang-2 expression and lower miR-122-5p levels were detected in ASCP with respect to PDAC **(A)**. Lower Tie-2, Ang-1 transcript levels and higher miR-21-5p, miR-27a-3p and miR-181a-5p were observed in ASCP with respect to PDAC **(B)**.

Comparing genes and miRNAs expression between ASCP and PDAC, a higher level of genes and lower level of miRNAs (or viceversa) were present in ASCP showing a more active angiogenetic pathways in ASCP compared to PDAC. As shown in the Figure 1A, lower levels (fold change = 0.27) of miR-122-5p and higher expression of VEGFA (fold change = 1.43), HIF1A (fold change = 1.46) and Ang-2 (fold change = 1.5) genes were observed in ASCP compared to PDAC subtype. Furthermore, lower Ang-1 (fold change = 0.73) and Tie-2 (fold change = 0.27) transcript levels and higher increases of miR-21-5p, miR27a-3p and miR-181a-5p (3.01 to 5.12- fold) levels were found in the rarest form of pancreatic carcinoma (Figure 1B).

**Figure 2:**
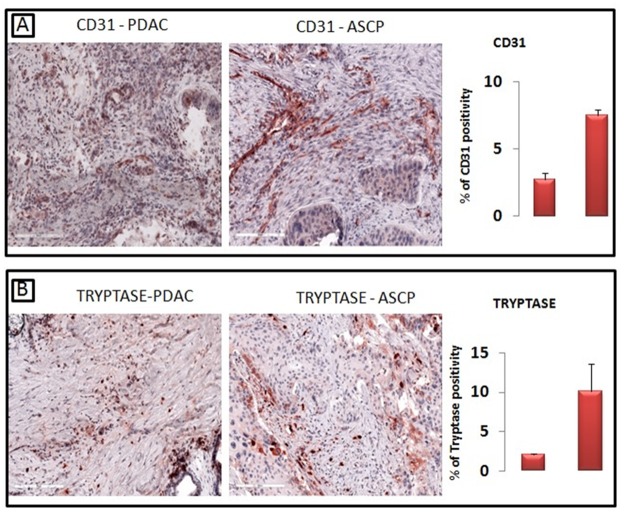
A greater number of CD31-positive microvessels are present in ASCP compared with PDAC, as confirmed by morphometric evaluation (A) Tryptase-posive mast cells are significantly higher in ASCP compared with PDAC, as confirmed by morphometric evaluation **(B)**.

These data seem to be supported by the evidence of a greater number of microvessels in ASCP with respect to PDAC as confirmed by morphometric evaluation (Figure 2A). The percentage area covered by vessels profiles was higher in the ASCP (7.3±0.9) than in the PDAC (2.7±1.1). Tryptase-posive MCs were also higher in ASCP (10.2±3.2) compared with PDAC (2.1±0.2) (Figure 2B).

## DISCUSSION

The angiogenesis has been well-studied in PDAC, in which the novel blood network results in response to hypoxia, although vessels appear compressed by the abundance of stroma [8]. The involvement of epigenetic mechanisms in the regulation of angiogenesis has been well-described in various tumors [10, 13]. MiRNAs are endogenous small non-coding RNAs that regulate gene expression at post-transcriptional level by binding to the 3′-untranslated region (UTR), 5′-UTR or coding sequences of target mRNA transcripts [5]. Increased evidences suggest a role for miRNAs in the control of PDAC blood network [8], but in this regard no data are available in ASCP. Due to its rare entity, limited data are available on ASCP and there are no guidelines for treating patients with this disease [14]. For this reason, there is a need to better characterize this rare disease in order to identify markers for the development and optimization of treatment. To our knowledge, this is the first study exploring the main pathways governing angiogenesis and their epigenetic regulation in ASCP. The expression genes of both angiopoietins and their receptor Tie-2, VEGFA, HIF1A and levels of miR-21-5p, miR-181a-5p, miR-122-5p and miR-27a-3p were analyzed. Among all miRNAs defined as “angiomiR” [15], an oncogenic role has been demonstrated for miR-21 in many solid tumors [16], including PDAC [6, 17]. In particular, the contribution of hypoxia in the increased expression of miR-21 and the capability of this miRNA to induce angiogenesis through HIF-1α expression have been reported by *in vitro* studies on pancreatic and prostate cancer cells, respectively [18, 19]. Interestingly, we observed higher levels of miR-21-5p in ASCP with an increased expression of HIF1A and VEGFA genes, suggesting an indirect regulation of them. More recently, it has been demonstrated the capacity of miR-21 to promote Tie-2 expression in brain microvascular endothelial cells [20]. Tie-2 is the tyrosine kinase receptor of ligands Ang-1 and Ang-2, whose expression is largely restricted to ECs. In particular, Ang-1 is an agonist for Tie-2 that mediates vessel maturation and maintenance of vascular integrity, whereas Ang-2 is associated with vascular regression and increased angiogenesis in the presence of VEGFA. Previously, it has been reported the ability of pancreatic stellate cells to improve angiogenesis by enhancing the levels of Ang-1 and its receptor Tie-2 mRNA in PDAC microenvironment [21, 22]. In the present study, according to our *in silico* analysis, lower Tie-2 and Ang-1 expression was found in ASCP, in which higher miR-21-5p, miR-27a-3p and miR-181a-5p levels were observed. No data are available about miR-27a-3p and miR-181a-5p involvement in angiopoietins/Tie-2 axis. However, oncogenic function in the regulation of angiogenesis process has been previously described for miR-27a-3p [23], whereas a role as hypoxia-regulated miRNA enhancing VEGF expression has been reported for miR-181a-5p [24]. Lower levels of Ang-1 and its cognate receptor Tie-2 suggest the possibility of an angiogenic process governed by other angiogenic factors in ASCP. In this regard, in addition to VEGFA and HIF1A, also increased levels of Ang-2 were observed in this rare malignancy with lower expression of miR-122-5p. Overexpression of Ang-2 correlated with poor prognosis in several tumors [25–27]. In the activated endothelium, the release of Ang-2 from Weibel-Palade bodies results in the improvement of ECs responsiveness to VEGF [28]. Both VEGF and tissue hypoxia have been shown to upregulate expression of Ang-2 that competitively inhibits binding of Ang-1 to the receptor Tie-2, enhancing VEGF-mediated angiogenesis. Deregulation of miR-122 has been described in several solid tumors, including PDAC [29]. To support our data, we observed a higher MVD in an ASCP case, as demonstrated by CD31 positive vessels distribution and tryptase-positive MCs with respect to PDAC. No data regarding vascular microenvironment in ASCP have been reported in the literature. Accordingly, a previous angiography-analyses of ASCP patients described these tumors as hypervascular compared to PDACs that are usually hypovascular tumors [30, 31]. Moreover, differently from PDAC, some recent analysis demonstrated that ASCP enhancement contrast pattern on computerized tomography shows persistence in the portal vein phase, suggesting a higher blood supply, although the correlation between the perfusion parameters and the immunohistochemical and molecular parameters was not evaluated [32–34]. It has been well-demonstrated that MCs contribute to the formation of novel tumor blood vessels through the release of tryptase, an active serine protease that represents the most powerful angiogenic mediators of these cells [35]. Another typical feature of PDAC is the presence of an intense fibro-inflammatory reaction with an abundant deposition of dense collagen types I and III bundles, hyaluronic acid and fibronectin that did not appear in ASCP, since triptase could cut them favouring angiogenesis.

Since ASCP is characterized by a poorly differentiated histology and a poorer clinical outcome compared to PDAC, the key role played by angiogenesis in tumor growth and metastasis in several cancers could partially justify its worse outcome.

For the first time, main pathways governing angiogenic process and their epigenetic regulation were investigated in ASCP with respect to PDAC. Given the low incidence of this malignancy, a better molecular and epigenetic characterization of ASCP may offer insight into potential therapeutic and diagnostic targets. Our evidence suggests a significant angiogenic activity in ASCP and a putative role of miR-21-5p, miR-181a-5p, miR-122-5p and miR-27a-3p in the epigenetic regulation of this process. Even if related to a single patient, our data support the need to evaluate this rare pancreatic cancer hystotype from a molecular profile with the aim to identify an antiangiogenetic therapeutic target.

## MATERIALS AND METHODS

### Patients

**Figure 3:**
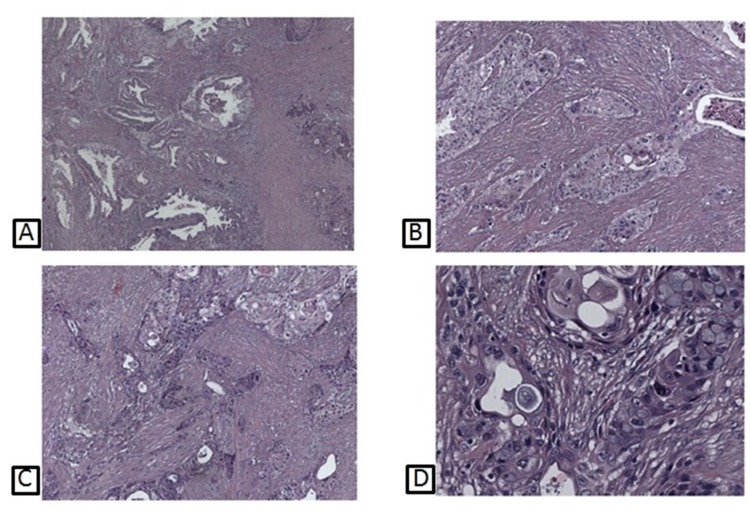
Histological pictures of different patterns of ASCP, showing prevalent adenocarcinomatous glands **(A)**; squamous neoplastic component **(B)**; a combination of both adeno- and squamous areas **(C)**; transition features between adenocarcinomatous elements, with interposed goblet cells, and squamous neoplastic islands **(D)**.

A 68-year-old man underwent a duodeno-cefalo-pancreatectomy for a pancreatic tumor with clinico-radiological suspicion for malignancy. The histopathological evaluation revealed the presence of a case of ASCP (Figure 3). Patient signed an informed consent form authorizing the research and all data have been processed with respect for privacy and anonymity.

### In silico analysis, genes and miRNA detection

TarBase v.6 [11] and MiRWalk v.2 [12] database were used to provide information about validated or predictive miRNAs-genes target interaction.

Total RNA was extracted from 2μm formalin-fixed, paraffin-embedded tissues sections using the RNeasy^®^ FFPE Kit (QIAGEN). For transcripts level analysis, 500 ng of total RNA were reverse transcribed using the High Capacity cDNA Reverse Transcription Kit, according to the manufacturer's protocol (Applied Biosystem). The ID assays used were the following: human VEGFA (Hs00900055_m1), human HIF1A (Hs00153153_m1), human Ang-1 (Hs00375822_m1), human Tie-2 (Hs00176096_m1) and human Ang-2 (Hs01048042_m1). RN18S1 (Hs03928985_g1) was used as the endogenous reference.

Briefly, for detection of miRNAs expression levels, 10 ng of total RNA were reverse transcribed using the TaqMan^®^ MicroRNA Reverse Transcription Kit and miRNA specific primers according to the manufacturer's protocol (Applied Biosystems), as previously described [36].

Quantitative real-time PCR was performed on the ABI Prism 7000 Sequence Detection System (Applied Biosystems) in accordance to the manufacturer's instructions (Applied Biosystems). MiRNAs and genes expression levels were calculated using ΔΔCt method after normalization with endogenous reference and PDAC as calibrator. All PCRs were performed in triplicate including no-template controls.

### Immunoistochemical evaluation of tryptase and CD31

Four μm paraffin-embedded tissues sections were retrieved on and incubated with antibodies against tryptase (DAKO, Glostrup, Denmark) and CD31 (Novocastra, Leica Biosystems, Nussloch, Germany) according to manufacturers’ instructions. The slides were then incubated with biotinylated anti-mouse Igs, peroxidase-conjugated streptavidin and diaminobenzidine (DAB). Counterstaining was performed with hematoxylin. Ten slides were scanned for each antibody using the whole-slide scanning platform Aperio Scanscope CS (Leica Biosystems, Nussloch, Germany), then were assessed with the Positive Pixel Count algorithm embedded in the AperioImageScope software and reported as a percentage of positivity, defined as the number of positively stained pixels on the total of pixels of the image.

## Abbreviations

ASCP: Adenosquamous carcinoma of the pancreas
PDAC: pancreatic ductal adenocarcinoma
MVD: low microvascular density
MCs: mast cells
Ang-1: angiopoietin-1
Ang-2: angiopoietin-2
Tie-2: receptor tyrosine kinase with immunoglobulin and epidermal growth factor homology domain-2
VEGFA: vascular endothelial growth factor A
HIF1A: hypoxia-inducible factor 1 alpha
microRNA: miRNA
ECs: endothelial cells
UTR: untranslated region
